# Impedance Spectroscopic Investigation of Proton Conductivity in Nafion Using Transient Electrochemical Atomic Force Microscopy (AFM)

**DOI:** 10.3390/membranes2020237

**Published:** 2012-06-06

**Authors:** Steffen Hink, Norbert Wagner, Wolfgang G. Bessler, Emil Roduner

**Affiliations:** 1 Institute of Physical Chemistry, University of Stuttgart, Pfaffenwaldring 55, Stuttgart D-70569, Germany; Email: s.hink@ipc.uni-stuttgart.de; 2 Institute of Technical Thermodynamics, German Aerospace Center, Pfaffenwaldring 38-40, Stuttgart D-70569, Germany; Email: norbert.wagner@dlr.de (N.W.); wolfgang.bessler@dlr.de (W.G.B.)

**Keywords:** Nafion membrane, electrochemical AFM, impedance spectroscopy, single pore proton flux

## Abstract

Spatially resolved impedance spectroscopy of a Nafion polyelectrolyte membrane is performed employing a conductive and Pt-coated tip of an atomic force microscope as a point-like contact and electrode. The experiment is conducted by perturbing the system by a rectangular voltage step and measuring the incurred current, followed by Fourier transformation and plotting the impedance against the frequency in a conventional Bode diagram. To test the potential and limitations of this novel method, we present a feasibility study using an identical hydrogen atmosphere at a well-defined relative humidity on both sides of the membrane. It is demonstrated that good quality impedance spectra are obtained in a frequency range of 0.2–1000 Hz. The extracted polarization curves exhibit a maximum current which cannot be explained by typical diffusion effects. Simulation based on equivalent circuits requires a Nernst element for restricted diffusion in the membrane which suggests that this effect is based on the potential dependence of the electrolyte resistance in the high overpotential region.

## 1. Introduction

Proton exchange membrane fuel cells (PEMFCs) represent a promising energy conversion technology for mobile and portable applications. The development of deeper insight into the functionality of PEMFC components calls for new experimental methods. One of the most important components is the proton exchange membrane which warrants the performance of the PEMFC by its inherently high proton conductivity, and its longevity via its chemical and thermal stability. Different structural models of Nafion, mainly based on small-angle x-ray and small-angle neutron scattering experiments have been developed. A model derived by Schmidt-Rohr fits best the observed experimental data and excludes several other models [1]. It proposes a structure consisting of bundles of parallel cylindrical water nanochannels embedded in a hydrophobic moiety. Although this model seems to be the most meaningful, all models are based on microphase separation in which proton conductivity originates in hydrophilic domains on the nanometer scale. This characteristic is related to the molecular structure of Nafion which has a hydrophobic backbone with side chains terminated by hydrophilic sulfonic acid groups [2]. 

For further investigations of the proton conducting domains, the so-called electrochemical atomic force microscopy (EC-AFM) was developed [3,4]. In this technique, a platinum coated AFM tip serves as a nanoscale cathode in a two-electrode assembly. It enables the detection of currents in the pA to nA range, with a spatial resolution of 10 nm, and dependent on the applied voltage and the relative humidity (RH) of the gas atmosphere. Some time-dependent experiments were performed, giving insight into the dynamics of the membrane dependent on the proton flux [5]. In first experiments with the tip placed on a proton conductive domain, a voltage step was applied between the two electrodes, giving rise to a transient current. By high resolution-imaging it was possible to directly visualize the proton conductive areas on the membrane [6]. Other work examined different proton exchange membranes (perfluorinated and hydrocarbon membranes) on a nanometer scale [7]. 

A variant of the AFM-based technique was reported by O’Hayre *et al*. [8]. These authors combined an AFM instrument with a potentiostat, conducting impedance measurements. Among other samples, Nafion was studied by an impedance mapping mode. Further work deduced a relation between the contact force and the contact area between a tip and a Nafion membrane [9]. This enabled quantitative impedance measurements on a nanoscale, based on nanoindentation experiments. 

Reaching a high spatial resolution with an AFM is difficult since the sample is strongly influenced by environmental parameters like temperature and RH. The resulting sample drift affects the resolution, and therefore a short measurement time is favorable. In the present, work, the setup of EC-AFM is extended to permit chronoamperometric experiments with high spatial and temporal resolution. The current response to a rectangular voltage step is measured as a function of time and converted to frequency space by Fourier transformation, allowing the impedance spectrum to be calculated and simulated using common equivalent circuits. For simplicity, the novel approach is tested by measurements of a hydrogen pump across a Nafion membrane as a model system. 

## 2. Experimental Procedures and Data Analysis

### 2.1. Instrumental Setup

**Figure 1:**
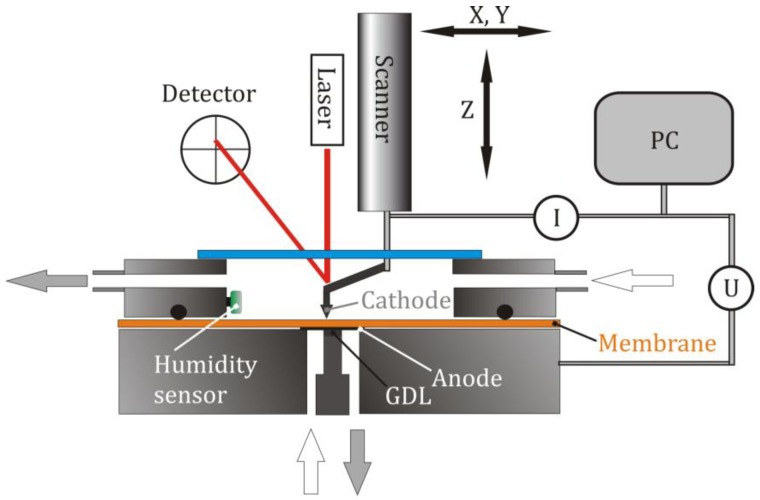
Experimental set-up: A membrane separates two environmental chambers with gas atmospheres which can be controlled independently but were identical for the present work. The upper side is sealed off by a perfluorosilicone cap in which the cantilever holder is embedded. The cantilever deflection is measured through a small glass window. An external PC runs the chronoamperometric experiments.

The experimental setup consists of an atomic force microscope (Dimension 3100, Veeco) equipped with a conductive AFM module, enabling the measurement of currents between the tip and the sample in the pA to nA range. The construction of the measurement cell is sketched in Figure 1. The one-side platinum coated membrane (Hispec 1000, Johnson Matthey) is mounted in the cell in such a way that two separate environmental chambers result. The electrical contact to the anode is provided by a gas diffusion layer (GDL). The lower chamber is included within the stage and sealed by the membrane itself. The upper chamber consists of a cylindrical disk with inlet and outlet for the gases. Towards the membrane it is sealed by an o-ring. The cantilever holder is fed through an elastic perfluorosilicone sealing cap (light blue) which ensures the functionality of the AFM. The laser beam for monitoring the cantilever deflection has access through a small glass disk in the cap. For control of the relative humidity a sensor is installed directly in the upper chamber. All measurements were performed at ambient temperature. During the measurements a hydrogen flow rate of 17.5 mL min^−1^ with the same RH was provided for each chamber. For the measurement of transients the existing experimental setup is extended by a signal access module (SBOB3, Veeco) which provides access to all electrical signals of the AFM-system. The chronoamperometric experiments were performed using a data acquisition card from National Instruments (PCIe-6361) which was controlled by the LabVIEW software (Version 10.2). The main features of the in-house written data acquisition software are an adjustable sampling frequency, a variable duration of the measurement and a freely selectable voltage step.

### 2.2. Sample Preparation

All samples were prepared from Nafion^®^ which was activated following a standard procedure [10]. First, the membrane was boiled in 5 wt. % H_2_O_2_ at 85 °C for 1 h and then rinsed with doubly distilled water for 15 min. Following this, it was placed in 0.5 M sulfuric acid at 85 °C for 1 h and subsequently rinsed again with doubly distilled water for 15 min. After drying in air, the membrane was coated on one side using a wet spraying preparation technique. The ink was prepared from platinum black (Hispec 1000, Johnson Matthey), doubly distilled water and Nafion Dispersion (10 wt. %, Aldrich) which was all mixed together and stirred for at least 12 h. The procedure leads to a catalyst loading of around 1 mg Pt per cm^2^ with 30 wt. % Nafion within the electrode. The coated membrane is stored in doubly distilled water for at least 24 h prior to use.

### 2.3. Fourier Transformation

The transient current and the corresponding voltage signals are transformed to the frequency domain by Fourier transformation. The temporal resolution of the signal traces plays an important role. The highest defined frequency (with amplitude and phase) of a discrete signal is given by the sampling theorem and is equal to or smaller than half of the sampling rate. The lower frequency limit is given by the reciprocal duration of the measurement [11]. These are theoretical limits, and a comparison with the characteristics of the system is necessary to estimate the limits of the real system (see below).

To calculate the impedance of the system, the voltage and the current signals, given by arrays of 10,000 points per second, are Fourier transformed (Equation (1)) using a numerical procedure described elsewhere [12]. 


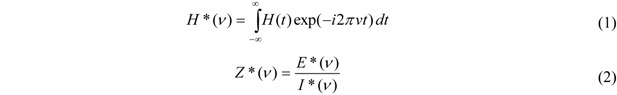


The function H(t) represents the time dependent voltage or current signal which is transformed to the complex function H*(ν) in the frequency domain. Subsequently, the impedance (Z*) is calculated by complex division according to Equation (2), where 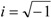. In the present work 100 points were calculated within each frequency decade.

## 3. Results and Discussion

### 3.1. Characterization of the System

**Figure 2:**
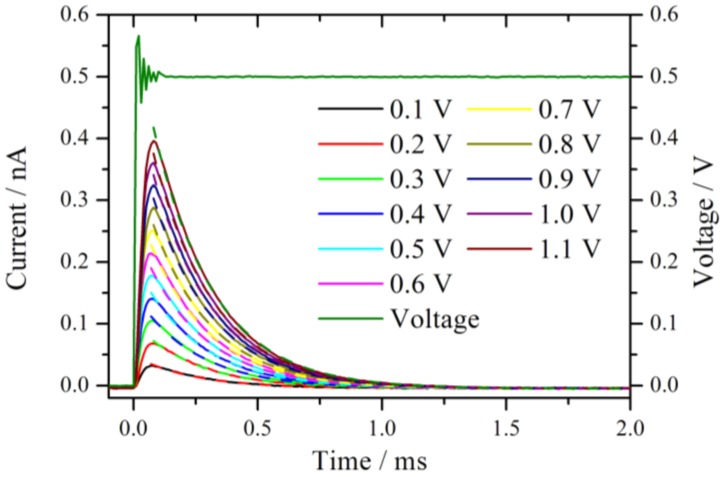
Current traces measured in a series by increasing the potential jump from 0 V to the corresponding value given in the diagram. The measurements were performed at the same position in a hydrogen atmosphere with 76% RH. The dashed lines represent the fitted exponential decay with a time constant of around 0.24 ms.

The AFM tip was placed on a non-conductive position of the Nafion 212 membrane in a hydrogen atmosphere at 78% RH. A series of voltage steps was applied and held for 5 s before it was set back to zero. The current and voltage signals were sampled with a rate of 100,000 s^−1^. The waiting time before the next experiment was several seconds. The first voltage step was from 0 V to 0.1 V, and the upper value was incremented by 100 mV for each subsequent measurement. Figure 2 shows an expansion of the measured current traces after the onset of the voltage step. The measured voltage from the set 0.5 V step is displayed as well. Before the voltage reaches the set value, a damped oscillation with an approximate duration of 100 µs is observed. This is the typical ringing behavior of a potentiostat [11] which limits distinctly the temporal resolution of the chronoamperometric experiments. The system is under strict potentiostatic control only after the oscillation has damped out. For a voltage step from 0 V to 1.1 V the duration of the ringing increases to 200 µs. However, as will be shown below, this oscillation is not the bottleneck of the system, rather, the time constant of the cell defines the lower temporal limit.

All current traces show a fast increase followed by an exponential decay to zero current. This is the typical behavior of an ideal polarizable electrode which can be represented by an equivalent circuit consisting of a capacitor connected in series with an Ohmic resistance. When a step voltage is applied to this circuit, the current response I(t) is entirely capacitive and described by an exponential decay with a time constant τ = R_S_∙C_D_ [13],

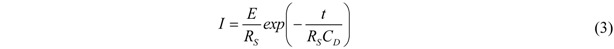


E represents the plateau voltage, C_D_ the capacity, and R_S_ reflects the Ohmic resistance of the solid electrolyte. The dashed lines in Figure 2 are the exponential fits to the current signals. The fits are calculated starting from the maximum current values. The time constants are 0.24 ms for the entire set of curves. At t ≈ 4τ the non-Faradaic current has dropped to 1.8% of the initial (E/R_S_) value. With the extracted value, τ = 0.24 ms, it takes at least 1 ms until meaningful information can be observed from this system in a chronoamperometric experiment. In other words, the cell constant of the system restricts the high frequency limit to 1 kHz, which is chosen as the upper limit for the calculation of the impedance spectra. 

To comply with this fact in accordance with the sampling theorem, the sampling rate for the subsequent measurements is chosen to be 10,000 points per second. 

The total exchanged charge is obtained by calculating the areas under the curves in Figure 2. Approximately, the charge scales linearly with the size of the voltage step, leading to an almost voltage independent mean capacitance of 1.13 × 10^−4^ nF. Assuming a typical geometric capacitance of 30 µF cm^−2^ for platinum, the area is calculated to 0.37 µm^2^. This corresponds to a radius of 346 nm if a circular contact area is assumed. As the radius of the tip is known to be about 10 nm, this contact area seems rather large. It is therefore likely that the observed capacitance is increased by other effects as for example pseudocapacitance due to hydrogen adsorption [14]. However, there is no evidence for a biexponential behavior in the curves of Figure 2.

The small size of this electrode has a pronounced influence on the characteristics of the electrochemical system. The measured currents are in the pA to nA range, which in principle corresponds to a very low IR drop or permits using highly resistive electrolytes giving a moderate IR drop. In any case the use of a two-electrode system is meaningful. Additionally, regarding the highly resistive electrolyte, the high temporal resolution of 1 ms is a result of the small size of the electrode, which has a small capacity C_D_ and lowers the time constant according to τ = R_S_∙C_D_.

### 3.2. Reproducibility of the Measurements

The reproducibility of the current-time traces were investigated by performing many measurement series for several experimental parameters. The aim was to verify a systematic behavior and find the optimum experimental parameters. Apart from the applied force between the tip and the sample and the RH of the gas atmosphere, the position on the sample and the recovery time between the measurements, which is a measure for the relaxation time of the membrane, were also varied. All measurements were carried out in a hydrogen atmosphere following a voltage step from 0.4 V to 0.5 V. The duration of a single measurement is 10.5 s whereby the voltage step was applied 0.5 s after the start. Five measurements were recorded for each set of parameters. The RH values were chosen to be 47%, 61% and 80%. For each RH value the measurements were taken at two different tip positions on the membrane so that a total of 6 different positions were investigated. On each position the force was varied from 10 nN to 30 nN and 50 nN, and also the time delay within the series of five measurements was varied from 10 s to 60 s and 180 s. Overall, 270 measurements were recorded. 

To permit a suitable comparison, the relative error based on the standard deviation of the data in this measurement series is determined in the following way. The arithmetic mean, 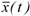, of each series is calculated according to Equation (4). More precisely, for each data point at time t the arithmetic mean value of all five measurements is determined. 0 ≤ *i* ≤ *N* denotes the number of the measurement within a series (*N* = 5):

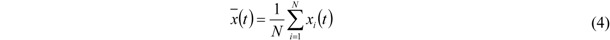


The relative error 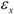, based on 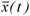 and corresponding to one standard deviation, is calculated from equation (5) for each measured curve of this series. Here *k* denotes the data points of the corresponding curve and *N* is the total number of data points:

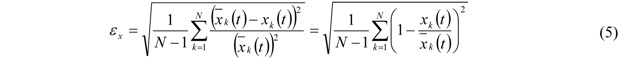


**Figure 3:**
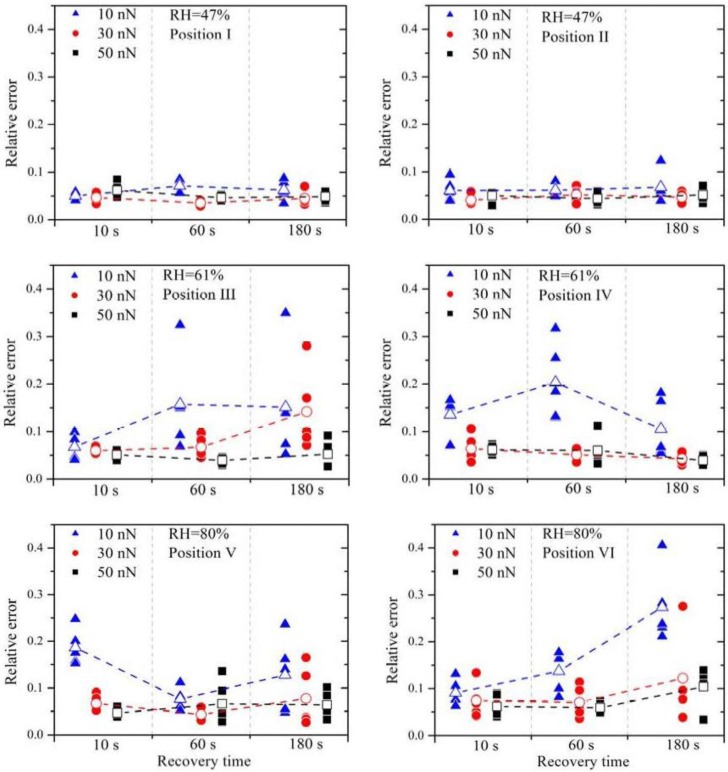
Relative errors, calculated from several measurement series, are plotted as a function of the applied force between the tip, the relative humidity of the gas atmosphere, the tip position on the sample and the recovery time between the different measurements. The open symbols represent the arithmetic mean of the five measurements.

The relative errors (filled symbols) and the arithmetic mean of the relative errors (open symbols) are plotted in Figure 3. It is obvious that a small contact force (*F* = 10 nN), marked by the blue triangles, leads almost always to the highest relative error, independent of RH. Between the contact force of 30 nN and 50 nN no distinct difference is observed. 

High contact forces can lead to damage of the membrane and also to high wear of the platinum catalyst layer on the tip. Hence, for the later experiments, the force was chosen to be 30 nN. 

The increase of the RH leads to much higher relative errors. At 47% RH all values of ε*_x_* are clearly less than 0.1, whereas at 61% RH a much higher scatter of the values is present. It is known that the water content in the membrane and the proton conductivity increase with RH [15]. Therefore, higher current signals are expected with higher RH, which is confirmed by these current measurement series. No significant change of the relative errors is observed by further increasing the RH.

The current signals from position V are a factor two or three higher than those from position VI, indicating that the local proton conductivity at position V is significantly higher. This difference reflects the inhomogeneity of the membrane surface and therefore the suitability of this method to obtain spatially resolved measurements. To obtain similar relative errors for position V and position VI, the deviations between the measurements have to be a factor two or three higher as well, indicating that the deviations scale with (proton) current. Due to the electro-osmotic drag a certain amount of water molecules is dragged along with the protons. If the proton current is higher, the water distribution within the membrane is much more strongly affected. It is therefore suggested that the growing perturbation of the water distribution within the membrane with increasing proton flux is the dominating origin of the relative error at higher RH. Experimentally, the influence of the relaxation of the membrane due to the perturbed water distribution can be investigated by varying the recovery time between subsequent measurements. Regarding the measurements with 30 nN and 50 nN, an overall higher relative error is observed with a recovery time of 180 s. During further experiments it turned out that a recovery time of 30 s or 60 s leads to the best results.

### 3.3. Measurements at a Proton Conducting Position

**Figure 4:**
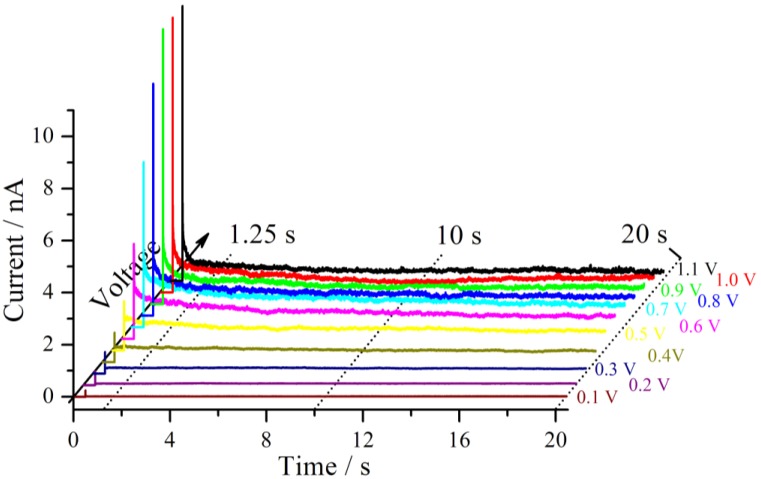
Starting at 0 V, different voltage steps were applied to a Nafion 117 sample in a hydrogen atmosphere at RH = 71% and an applied force of 30 nN between the sample and the tip. The resulting current traces are shown. The break between the measurements was 30 s.

A series of measurements was conducted at a fixed tip position on a Nafion 117 membrane in a hydrogen atmosphere with an RH of 71%. For the first measurement, the voltage step was applied from 0 V to 0.1 V, whereas for each subsequent measurement the plateau voltage is incremented by 100 mV. According to the reproducibility of the measurements discussed above, the delay time between the measurements was chosen to be 30 s and the tip was pressed onto the sample with a force of 30 nN. The corresponding current traces are shown in Figure 4. The voltage step is applied after 0.5 s of the start of the measurement.

**Figure 5:**
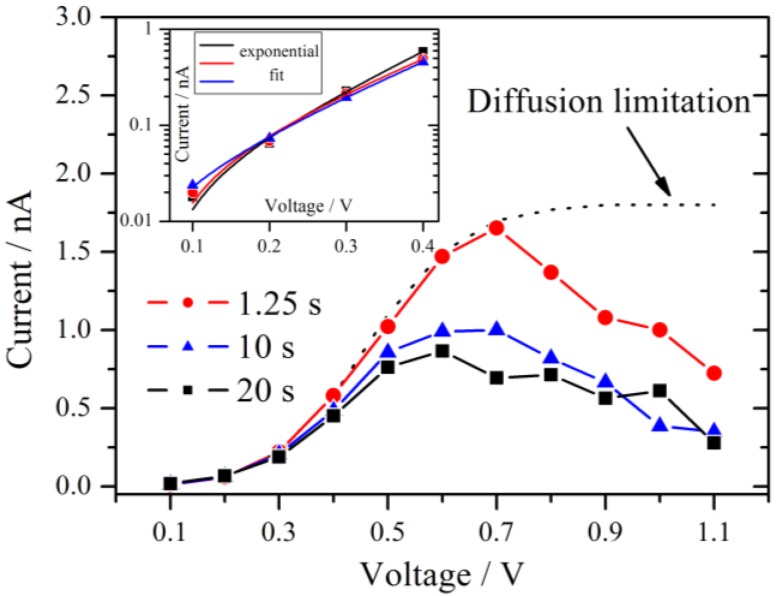
Current-voltage polarization curves derived at three different times after the beginning of the measurement as indicated in Figure 4. A maximum is observed near 0.6 V–0.7 V. The typical diffusion limitation is indicated by the dashed line. The inset shows a Tafel plot of the overpotential region <0.5 V with the corresponding exponential fits.

Shortly after the voltage step, a strong increase of the current is observed for each measurement, which is mainly based on the capacitive current of the cell. For the first 7 voltage steps up to the terminal voltage of 0.7 V, each subsequent current trace increases further. This behavior may be better seen if the polarization curves of this measurement series are extracted. Therefore different profiles at different delay times parallel to the voltage axis from Figure 4 are formed [16]. For three different times, the first one at 1.25 s after the beginning of the measurement, the second after 10 s and the third one after 20 s, these plots are shown in Figure 5. 

From 0.1 V to at least 0.4 V all three curves follow an exponential increase. According to the theory of chronoamperometric experiments [13], the current increases exponentially due to Butler-Volmer kinetics in the low overpotential region.

This is confirmed by the inset of Figure 5 which shows typical Tafel plots from 0.1 V to 0.4 V. For very small overpotentials around 0.1 V the characteristic deviation from the Tafel behavior is observed.

If the overpotential is very high in an electrochemical system with an aqueous electrolyte, the maximum current at long times reaches the diffusion limitation. This theoretical limiting current is independent of the applied voltage and is drawn schematically as a dotted line in Figure 5. For the present experiments, the current decreases if the voltage step is increased above 0.7 V, which is not consistent with the theory of chronoamperometric experiments. A maximum of the current signal is passed at an applied voltage of 0.6 V–0.7 V. Such an effect is unusual but was observed previously in the steady-state polarization curves of a PEMFC under specific conditions. It was attributed to the dependence of the Nafion membrane conductivity on cell potential [17]. The fact that this behavior is also observed for the hydrogen pump of the present experiment excludes that oxygen reduction reaction is the source of this effect. It is noteworthy that for a high RH of 89% the maximum in the current has almost vanished and an approximate diffusion limited behavior is observed.

**Figure 6:**
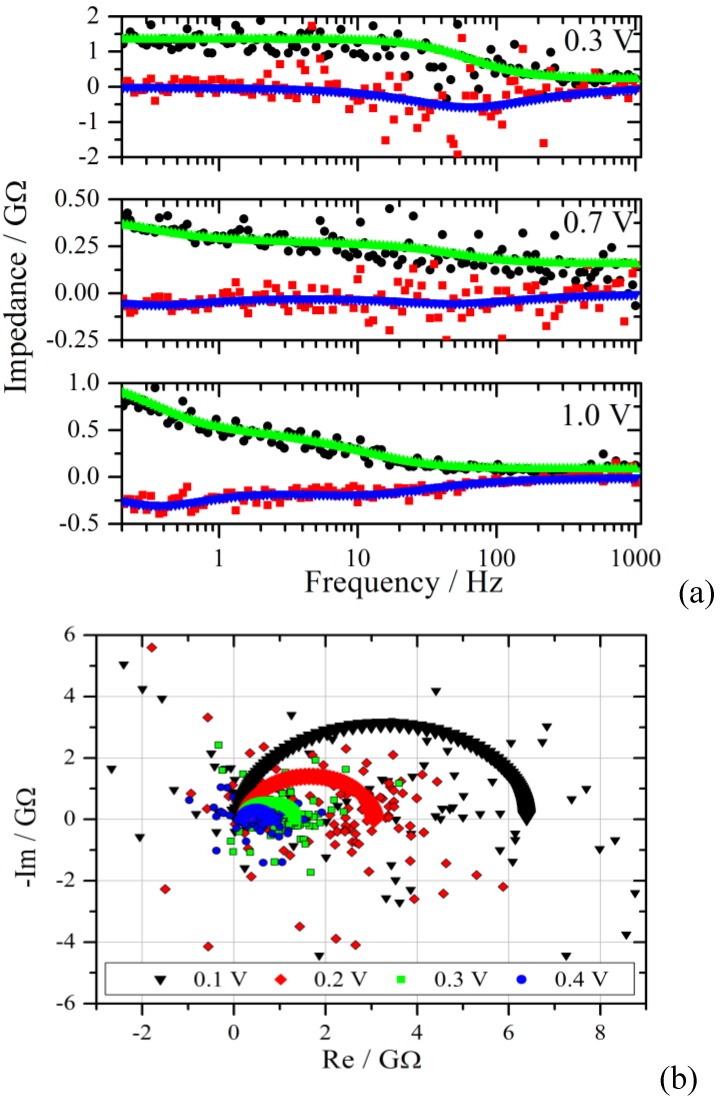
(**a**): Bode plot of the impedance spectra from the measurements of Figure 4 with a voltage step from zero to 0.3 V, 0.7 V and 1.0 V. The upper curves are the fits of the real part to the experimental data (black dots), the lower curves and red squares represent the imaginary part. Note the different Y-scales for the impedance; (**b**): Nyquist diagram of the experimental and the simulated impedance spectra for the voltage steps given in the graph.

The polarization curves selected in Figure 5 provide only a limited insight into this system. Therefore, a further way of analysis is chosen. The impedance spectra are calculated from the current traces and the corresponding voltage traces. As already discussed above it takes about 1 ms before meaningful information can be obtained from this system. This fact defines the high frequency limit for the Fourier transformation to 1 kHz. According to the measurement duration of 20 s, the theoretical lower frequency limit corresponds to 0.05 Hz. During the analysis of the data it turned out that the lower limit shifts to a higher value of 0.2 Hz, which seems to be rooted in the transformation of the discrete signals. The impedance spectra are calculated in the frequency range from 0.2 Hz to 1 kHz and shown in Figure 6 for voltage steps to 0.3 V, 0.7 V and 1.0 V.

As can be seen from Figure 4 and Figure 5, regions with different characteristics were chosen. In Figure 6 the real and the imaginary part of the transformed data are plotted together with the simulated spectra as a function of frequency. The scatter of the calculated impedance decreases with increasing voltage step, which is due to the improved signal-to-noise ratio from the experiment. 

The measurement with a voltage step to 0.3 V shows the typical behavior for a parallel RC-circuit. This behavior is representative for the voltage range from 0.1 V to 0.4 V. In the Nyquist plane the RC circuit manifests itself as a semi-circle with a radius that decreases with increasing potential for a Faradaic reaction. The simulated and experimental spectra of this voltage range are shown in the lower graph of Figure 6. Although there is good agreement in the Bode diagram, the scatter in the Nyquist plot is much larger. This effect is rooted in the superposition of the scatter from the real and imaginary part of the impedance.

**Figure 7:**
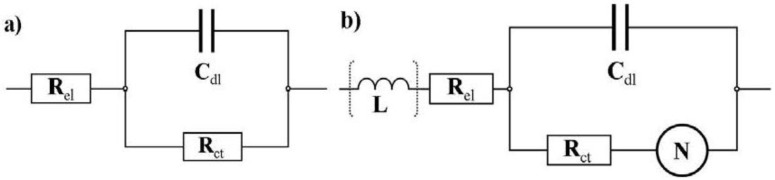
Equivalent circuits used for the simulations of the impedance spectra obtained from the data of Figure 4. (**a**) The RC circuit is used for the simulation of the impedance spectra with low overpotential; (**b**) The equivalent circuit is similar to the Randles circuit but the Warburg impedance is replaced by a finite-length diffusion element (Nernst diffusion element). This circuit is used for the moderate and high overpotential region.

A distinctly different characteristic is observed for the voltage range between 0.8 V and 1.1 V. The impedance starts to increase in the low frequency region, as can be seen exemplarily in Figure 6 for 1.0 V, indicating diffusion as the limiting process. The spectra in the range with low overpotential are simulated by using an RC circuit in series with an Ohmic resistance representing the electrolyte resistance R_el_, shown in Figure 7(a). In the moderate and in the high overpotential region a modified Randles circuit, shown in Figure 7(b), is used. This equivalent circuit consists of a capacitor in parallel with an Ohmic resistance R_ct_ representing the charge transfer resistance for the electrochemical reaction here mostly at the cathode, and a Nernst diffusion element *N* which is, in contrast to the Warburg diffusion element, limited to very low frequencies due to a finite-length restricted diffusion.

The Nernst diffusion is controlled by the Warburg coefficient W and the Nernst constant *k_N_* which is defined as 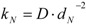 with the diffusion coefficient D and the thickness of the Nernst layer *d_N_*. This loop is in series with an Ohmic resistance which, as in the other equivalent circuit, represents the electrolyte resistance R_el_. The inductive behavior which is observed in the high frequency region for some spectra is simulated with an inductor L in series with R_el_. A complex non-linear least square regression algorithm is used for the fitting procedure.

**Figure 8:**
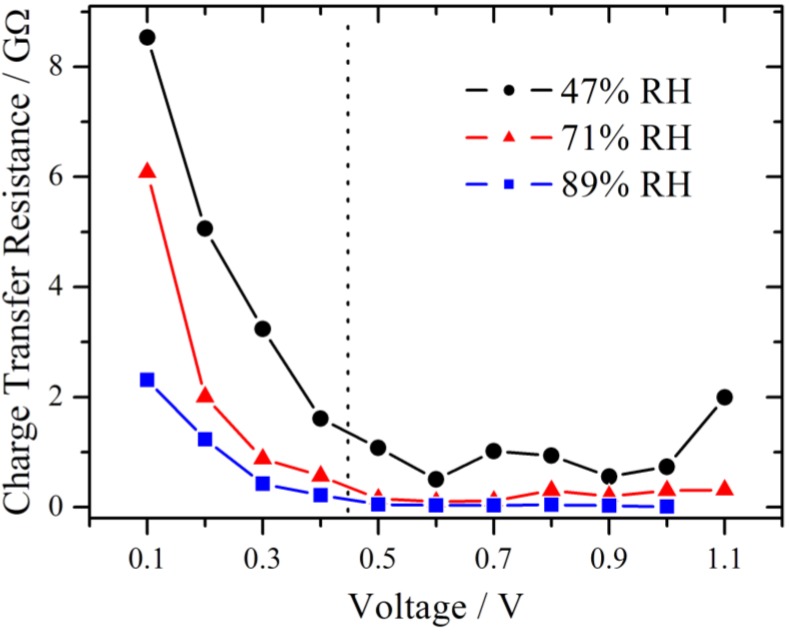
Simulated charge transfer resistance as a function of the voltage at different relative humidities, showing a pronounced decline with increasing voltage. The charge transfer resistance is smaller at higher relative humidity. In the section to the left of the dotted line the RC-circuit and to the right the modified Randles-circuit is used.

The results for the charge transfer resistance from the above measurement series (RH = 71%) are plotted in Figure 8 together with those from series with lower and higher RH. Due to the limit of the amplifier only measurements up to 1.0 V were available for 89% RH. From theory of electrochemical impedance spectroscopy, a strong dependence of R_ct_ from the potential is expected in the kinetically controlled region [18]. In the lower voltage range, the charge transfer resistance decreases strongly in an exponential manner for each of these three different RH values, suggesting that the charge transfer kinetics is the dominating process, as it was already concluded from Figure 5. Over the whole investigated potential range, an influence of the RH is observed. The value of the charge transfer resistance drops with increasing RH.

The impair of the performance of the oxygen reduction reaction was reported for a temperature dependent study of a proton exchange membrane fuel cell [19]. This was derived from the increase of the charge transfer resistance at the cathode with increasing temperature. Among other things it was discussed that the water content in the catalyst layer, especially in the Nafion ionomer, decreased, leading to an insufficient proton supply. In the present work, the RH is varied directly and the same behavior of an increase of the charge transfer resistance is observed. Although, the gas flow is identical for all measurement series, a small impact due to hydrogen partial pressure differences cannot be fully excluded. 

The simulated capacity shows little variation in the low overpotential region and reaches values of a few pF independent of the RH. For an overpotential >0.5 V up to 208 pF are observed for 89% RH, which is indicative of pseudocapacitance. 

**Figure 9:**
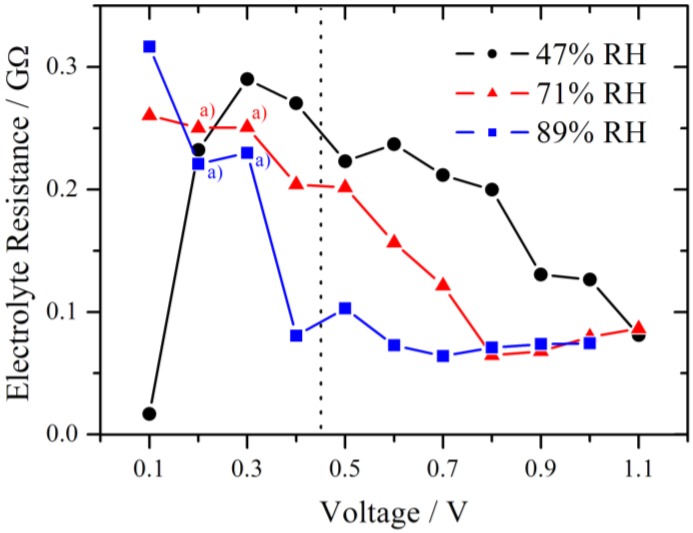
Simulated electrolyte resistance plotted for different relative humidities as a function of the voltage step. The dotted line marks the change from the RC-circuit (left side) to the modified Randles circuit (right side) used for the simulation. With increasing RH the electrolyte resistance decreases. a) indicates R_el_ values which were fixed during the simulation of the impedance spectra.

In Figure 9 the simulated electrolyte resistance is plotted for different RH as a function of the voltage step. As expected, R_el_ decreases with increasing RH due to higher proton conductivity within the membrane. Another observed effect is the declining tendency of R_el_ with increasing voltage step. Typically the increase of the electrolyte resistance with increasing current density is reported for Nafion in a fuel cell and attributed to a dehydration of the membrane at the anode side [20]. However, in this experiment the current is in the nA range. This corresponds to a very high current density for the small cathode but to a low current density for the large anode, and therefore only a slight change of the water distribution is expected at the anode. 

Although the samples were conditioned using a common procedure, internal reorganization of the local membrane structure due to the proton flux is possible. This may lead to an improved water distribution within the membrane and in the end to a lower electrolyte resistance. For 89% RH in the high overpotential region Rel is almost constant near 75 MΩ. This level is also reached at high overpotential for 47% RH (1.1 V) and 71% RH (0.8 V), respectively, and it seems to be the lower limit for these experimental conditions.

**Figure 10:**
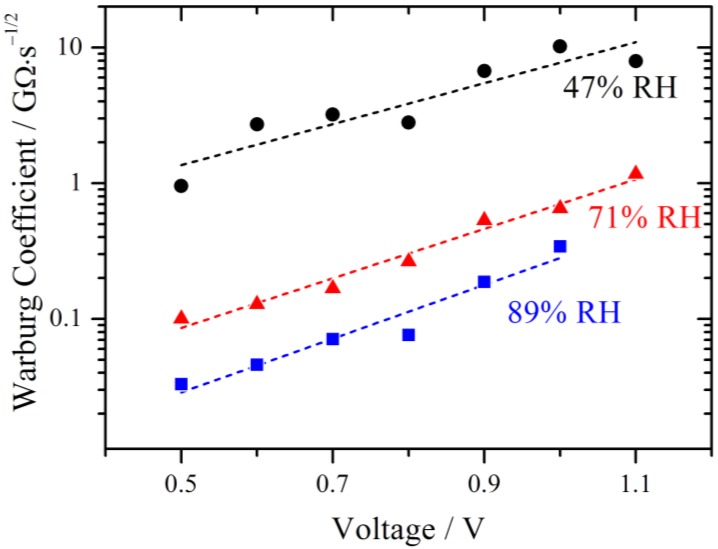
Simulated Warburg coefficients W plotted against the voltage for different relative humidities. With increasing RH the diffusion limitation decreases. An exponential increase with overpotential is observed as indicated by the fits (dashed lines).

The fitted values of the Warburg coefficient are plotted on a logarithmic scale against the voltage in Figure 10. It is obvious that the diffusion becomes more and more restrictive with increasing voltage.

The Warburg coefficient decreases with increasing relative humidity, indicating that the diffusion is less restrictive with higher RH. According to the model of Schmidt-Rohr [1] this behavior may be explained by a better connection of the parallel water cylinders within the membrane, leading to better pathways for the protons to diffuse. From a theoretical point of view this may be explained by a strong increase of proton conductivity with increasing water activity [21]. The limiting current due to bulk diffusion is sketched in Figure 5 and it is clear that the decreasing behavior of the current with increasing potential cannot be explained with a typical diffusion approach. As already mentioned above, such characteristics have been described for a PEMFC by a potential dependent membrane resistance. The proton migration between adjacent sulfonate groups on the surface of the pores of the membrane was discussed as a reason for this behavior [17]. The authors reported an exponential relation between the voltage and the cell polarization which they were able to relate to the conductivity of the membrane and which was also valid for polarization curves without a maximum current.

Such an exponential relation is also observed for the Warburg parameters in Figure 10 and elucidated by the linear fits (dashed lines). This is very remarkable, as the derivation of the Nernst diffusion element is based on homogenous bulk diffusion. It is therefore assumed that in this potential range the potential dependent transport of protons is the restrictive process. 

Another interesting fact is that the Nernst coefficient *k_N_* reaches values between 0.2 s^−1^ and 3.2 s^−1^ for all investigated RH. A diffusion coefficient can be calculated from the definition of *k_N_*. Based on the thickness of Nafion 117 of around 175 µm and *k_N_* = 3.2 s^−1^ D is calculated to be 0.98℘10^−5^ cm^2^ s^−1^. This value is very close to the reported diffusion coefficients for water in fully humidified Nafion [22] and supports that it is meaningful to use the Nernst impedance for describing the proton transport within the membrane. 

Although only absolute values are presented in this work, it should be noted that area-specific values can be obtained by normalizing with the contact area of the tip and the sample. Using the method presented by O’Hayre *et al*. [9] the contact area can be estimated based on nanoindentation experiments.

## 4. Conclusions

An extension of the EC-AFM technique is developed and shown to be suitable to measure impedance spectra with good quality and high spatial resolution. In view of the acquired frequency range, this method is much faster than frequency-domain impedance techniques. A short measurement time is necessary if the system is strongly sensitive to external influences such as temperature and relative humidity and thus susceptible to drift. The characterization of the system reveals optimized parameters for the measurement technique. The limits of a meaningful Fourier transformation of the current and voltage traces are determined to range from 0.2 Hz to 1 kHz. 

The simulations of the impedance spectra reveal detailed insight into the system. The local impedance is restricted by the charge transfer resistance in the low overpotential region. A decreasing current in the high overpotential region is observed in the polarization curves which were extracted from the current transients measured at different RH. There is evidence that this originates from a potential dependent electrolyte resistance, which is adequately described by a Nernst impedance element for restricted diffusion.
